# Prevalence of Hypertension and Its Associated Factors among Indonesian Adolescents

**DOI:** 10.1155/2020/4262034

**Published:** 2020-09-16

**Authors:** Andra Kurnianto, Deni Kurniadi Sunjaya, Fedri Ruluwedrata Rinawan, Dany Hilmanto

**Affiliations:** ^1^Faculty of Medicine, Universitas Sriwijaya, Palembang, Indonesia; ^2^Department of Public Health, Faculty of Medicine, Universitas Padjadjaran, Bandung, Indonesia; ^3^Division of Pediatric Nephrology, Department of Child Health, Faculty of Medicine, Universitas Padjadjaran, Bandung, Indonesia

## Abstract

**Background:**

Given that hypertension in adulthood has its onset in childhood, it is not surprising that the prevalence of hypertension among adolescents has also increased in recent years. However, there are limited data on the prevalence of hypertension and also the new AAP guideline has not yet been applied to the Indonesian adolescent population. Thus, this study aimed to evaluate the prevalence of hypertension using the new AAP guideline and to assess the occurrence of its associated factors among Indonesian adolescents.

**Methods:**

This was a cross-sectional study conducted at twelve senior high schools in Palembang, South Sumatera, Indonesia, from June to December 2019. The study included adolescents aged 13 to 18 years old. Anthropometric measurements were obtained. Multiple logistic regression was used to assess the risk factors most associated with hypertension among adolescents, and then an equation model was created. The prevalence of hypertension was evaluated, together with several factors such as age group, sex, ethnicity, family history of hypertension, nutritional status, physical activity, perceived stress, sleep duration, nutritional intake, and smoking.

**Results:**

In total, 1200 adolescents aged 15.9 ± 0.99 years were evaluated. The prevalence of hypertension and elevated blood pressure among adolescents was 8% and 12.2%, respectively. There were significant associations between sex, family history of hypertension, hypertensive father, nutritional status, physical activity, perceived stress, and hypertension among Indonesian adolescents (*p* < 0.05). Stress was the most powerful risk factor of hypertension with an odds ratio of 5.83 (95% confidence interval 2.91–11.6).

**Conclusions:**

Nowadays, the prevalence of hypertension among Indonesian adolescents is quite high. This may be caused by lifestyle or behavior changes among adolescents. Sex, family history of hypertension, nutritional status, physical activity, and perceived stress influenced the 27% hypertension prevalence rate among Indonesian adolescents, particularly in Palembang, South Sumatera. In order to decrease the prevalence of hypertension in adults, concern about lifestyle or behavior changes and hypertension among adolescents should be given.

## 1. Introduction

Hypertension is the most common noncommunicable disease, which persists as a significant health risk globally [1, 2]. In the last decade, its prevalence rate has increased by 5.2% worldwide [2, 3]. In Indonesia, the prevalence rate of hypertension has dramatically increased from 25.8% in 2013 to 34.1% in 2018 [4, 5]. Blood pressure (BP) levels in adulthood have been found to be significantly correlated with the BP levels in childhood [6]. As a predictor of hypertension in adulthood, the BP level during adolescence is more reliable than that during childhood [7]. The American Academy of Pediatrics (AAP) issued a new clinical practice guideline in 2017 for defining hypertension in children and adolescents that aligns with the new American Heart Association (AHA) and American College of Cardiology (ACC) guidelines [8]. Unfortunately, the new AAP guideline has not yet been applied to the Indonesian adolescent population. Given that hypertension in adulthood has its onset in childhood, it is not surprising that the prevalence of hypertension among adolescents has also increased in recent years [9, 10]. However, there are limited data on the prevalence of hypertension and its associated factors among Indonesian adolescents particularly in Palembang, South Sumatera. Thus, this study aimed to evaluate the prevalence of hypertension using the new AAP clinical practice guideline and to assess the occurrence of its associated factors among Indonesian adolescents.

## 2. Methods

### 2.1. Subjects

**Figure 1:**
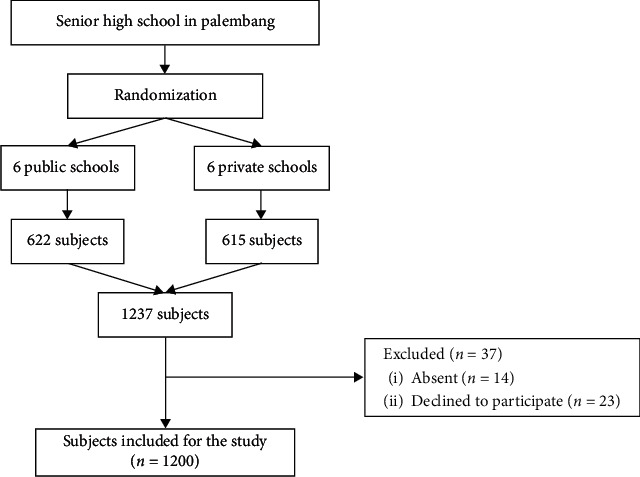
Study flow.

This multicenter cross-sectional study was conducted from June to December 2019 at twelve senior high schools in Palembang, South Sumatera, Indonesia. The subjects were recruited using multistage random sampling. The selection of senior high schools, consisting of public and private, was based on cluster random sampling and then continued with simple random sampling from the total number of students. The study was approved by The Health Research Review Committee of Mohammad Hoesin Central Hospital and Sriwijaya University as well as The Research Ethics Committee Universitas Padjadjaran, Bandung. The study was conducted in accordance with National Ethical Guidelines on Health Research and its Supplements. A written informed consent from parents and verbal ascent of each study participant were obtained prior to recruitment. Adolescents aged 13 to 18 years old who had no congenital abnormalities and medication that potentially increases or decreases BP, had their BP and anthropometry measured, and completed all questionnaires were included in the study. The study flow is presented in Figure 1.

### 2.2. Study Protocol

The hypertension criteria used in this study were based on the new AAP clinical practice guideline for adolescents aged ≥ 13 years old (normal BP: <120/<80 mmHg; elevated BP: 120/<80 to 129/<80 mmHg; Stage 1 HTN: 130/80 to 139/89 mmHg and Stage 2 HTN: ≥140/90 mmHg) [8]. The BP level was measured using Riester standard clinical aneroid sphygmomanometer and Littmann Classic II stethoscope with the subject in a sitting position and his/her right arm outstretched on the table. The measurement was performed thrice with a 5-minute interval and average when classifying BP. Body weight measurement was done using Seca digital scale with a capacity of 200 kg and accuracy of 0.1 kg. Body height was measured using Seca microtoise with a capacity of 200 cm and an accuracy of 0.1 cm. The subjects were asked to take off their footwear and hair accessories at the time of measurement.

The nutritional status or body mass index-for-age was assessed using World Health Organization (WHO) growth charts (Software WHO AnthroPlus version 1.0.4) and classified as obese (above +2 SD or more than or equal to the 97th percentile), overweight (above +1 SD or 85th to less than the 97th percentile), and normoweight (above −2SD to +1 SD or 15th to less than the 85th percentile) [11]. WHO AnthroPlus is a software for the global application of the WHO Reference 2007 for 5–19 year olds to monitor the growth of school-age children and adolescents [11].

Physical activity level was obtained using the Physical Activity Questionnaire for Adolescents (PAQ-A) and classified as less active (1 to 2 points) and active (3 to 5 points) [12]. PAQ-A is a self-administered 7-day recall questionnaire that assesses participation in different physical activities, as well as activities during physical education classes, during lunch breaks, after school, in the evenings, and at weekends [12]. Perceived stress was determined using the Perceived Stress Scale (PSS) and classified as perceived stress (14 to 40 points) and not perceived stress (0 to 13 points) [13]. PSS is the most widely used tool for measuring the perception of stress, with 10 items asking about feelings and thoughts during the last month [13].

Dietary intake data were obtained using three consecutive 24-hour dietary recalls [14] and defined using Recommended Dietary Allowances (RDA) for Indonesian adolescents aged 13 to 18 years old based on Indonesia Ministry of Health [15]. The 24-hour dietary recall method provides comprehensive quantitative information on individual diets by asking about food and beverages consumed during the previous 24-hour period [14]. Sodium intake was classified as high (more than 1.5 grams) and moderate (1.5 grams), fat intake classified as high (male: more than 89 grams; female: more than 71 grams) and moderate (male: 83 to 89 grams; female: 71 grams), fiber intake classified as low (male: less than 35 grams; female: less than 30 grams) and moderate (male: 35 to 37 grams; female: 30 grams), potassium intake classified as low (less than 4.7 grams) and moderate (4.7 grams), calcium intake classified as low (less than 1.2 grams) and moderate (1.2 grams) [15]. Family history of hypertension, smoking, and sleep duration were obtained using a self-report questionnaire. Sleep duration was classified as inadequate (less than 7 hours per night) and adequate (more than or equal to 7 hours per night) [16]. All the validated questionnaires were in Indonesian language (*bahasa*).

### 2.3. Statistical Analysis

The frequency distribution, mean, standard deviation, odds ratio, and 95% confidence interval were used for descriptive analysis of the data. Variables with *p* value smaller than 0.25 can be included for further multivariable analysis. Multiple logistic regression was used to assess the risk factors most associated with hypertension among adolescents, and then an equation model was created. The significance level was set at *p* < 0.05.

## 3. Results

The sociodemographic characteristics and lifestyle risk factors of the subjects are presented in Tables 1 and 2. There were 1200 adolescents consisting of 442 (36.8%) males and 758 (63.2%) females. Most of them in the midadolescence, that is, between 14 and 16 years old (82.8%). The ethnicity of a majority of the subjects was Palembangnese (72.7%), and only 37% had a family history of hypertension. Obese status and overweight status were found in 9.7% and 13% of subjects, respectively. Fifty percent of the subjects were less active, 31.8% had perceived stress, and 78.3% had inadequate sleep duration. The nutritional intake of the subjects consisted of low-fiber, low-potassium, and low-calcium diet (99.3%, 99%, and 98%, respectively). Only 3.3% of the subjects were smokers; 47.3% had a smoking father and 1.3% had a smoking mother.

The prevalence rates of hypertension and elevated BP among Indonesian adolescents in Palembang were 8% (7% stage 1 and 1% stage 2) and 12.2%, respectively (Table 3) with the average value of systolic BP 109 ± 10.6 mmHg and diastolic BP 72 ± 8.5 mmHg.  Table 4 shows blood pressure levels according to sex characteristics.

Table 5 presents the significant association between sex, family history of hypertension, hypertensive father, nutritional status, physical activity, perceived stress, and hypertension among Indonesian adolescents (*p* value < 0.05). Variable hypertensive mother and smoking were also included for further multivariable analysis (*p* value < 0.25).

Table 6 shows the final model of hypertension prevalence consisting of sex, family history of hypertension, nutritional status, physical activity, and perceived stress. The equation for hypertension prevalence was formulated. Hypertension prevalence = −6.909 + 1.377 (Sex) + 0.994 (family history of hypertension) + 0.787 (nutritional status) + 1.223 (physical activity) + 1.763 (perceived stress). The aforementioned variables were found to influence the 27% hypertension prevalence rate among Indonesian adolescents in Palembang, South Sumatera.

## 4. Discussion

In this study, the prevalence rate of hypertension among adolescents in Palembang, South Sumatera was 8%, which consisted of 7% stage I hypertension and 1% stage II hypertension. Our results indicated that the prevalence rate of hypertension in Palembang, South Sumatera, was higher than that of the Basic Health Research [4] in Indonesia and the study by Silva and Farias [17] in Brazil (5.3% and 7.4%, respectively) but lower than that of the study among adolescents by Pardede et al. [18] in Central Jakarta, Indonesia, and Bell et al. [19] in Houston, Texas, USA (9.6% and 11.6%, respectively). Pardede et al. [18] evaluated the prevalence of hypertension among Indonesian adolescents in Central Jakarta using different hypertension criteria based on the Fourth Report on the Diagnosis, Evaluation, and Treatment of High Blood Pressure in Children and Adolescent. The hypertension criteria used in this study were based on the new AAP clinical practice guideline that aligns with the new American Heart Association (AHA) and American College of Cardiology (ACC) guidelines [8]. Hence, blood pressure from childhood to adulthood can be traced.

With regard to sociodemographic factors, male adolescents had a 3.96 times higher risk of hypertension than female (*p* < 0.05). Even though the number of female adolescents was much higher in general population, the prevalence of hypertension among males was higher than female adolescents, which were 4.6% (3.8% stage 1 and 0.8% stage 2) and 3.4% (3.2% stage 1 and 0.2% stage 2), respectively (Table 4). The adolescents with a family history of hypertension had a 2.7 times higher risk of hypertension than those without a family history of hypertension (*p* < 0.05). These findings were consistent with those of several previous studies. According to the results of the studies by Silva and Farias [17] and Singh et al. [20], male subjects and adolescents with a family history of hypertension significantly developed hypertension more than female subjects and adolescents without a family history of hypertension.

In terms of lifestyle risk factors, it appeared that the risk of hypertension was higher among adolescents with an obese/overweight nutritional status who were physically less active than normoweight and physically active adolescents (2.19 and 3.39, respectively). These results are in accordance with those of several other studies. The study by Singh et al. [20] also showed that obese/overweight and less active adolescents more likely had hypertension than normoweight and physically active adolescents. Sleep duration, smoking, and nutritional intake such as high-sodium and high-fat diet as well as low-fiber, low-potassium, and low-calcium diet surprisingly did not appear to be contributory to the risk factors in this group. This may be caused by several factors; that is, only a small number of adolescents were smoking and eating a high-sodium and high-fat diet (3.3% and 6.7%, respectively).

Multivariate analysis was used to assess the strongest risk factors that have a significant association with the occurrence of hypertension among adolescents. This study found, in agreement with the study by Shipp et al. [21] in South Texas, that stress was the most powerful risk factor of hypertension with an odds ratio of 5.83 (95% confidence interval 2.91–11.6) although only a small number of adolescents (31.8%) responded to be under some form of stress, termed as perceived stress. Stress may increase unhealthy coping behaviors, such as inadequate sleep; excessive food consumption; consumption of foods high in salt, sugar, and saturated fat; smoking; or consumption of alcohol that are associated with hypertension among adolescents [22].

In conclusion, the prevalence of hypertension among Indonesian adolescents is currently high. This may be caused by lifestyle or behavior changes. Sex, family history of hypertension, nutritional status, physical activity, and perceived stress influenced the 27% hypertension prevalence rate among Indonesian adolescents in Palembang, South Sumatera. In order to decrease the prevalence of hypertension in adults, concern about lifestyle or behavior changes and hypertension among adolescents should be given.

## Figures and Tables

**Table 1:** Sociodemographic characteristics.

Characteristics	*N* (%)
*Age (years old)*	
14	198 (16.4)
15	416 (34.7)
16	380 (31.7)
17	206 (17.2)

*Sex*	
Male	442 (36.8)
Female	758 (63.2)

*Ethnicity*	
Palembang	872 (72.7)
Others	328 (27.3)

*Family history of hypertension*	
Present	444 (37)
Absent	756 (63)

*Hypertensive father*	
Yes	260 (21.7)
No	940 (78.3)

*Hypertensive mother*	
Yes	248 (20.7)
No	952 (79.3)

**Table 2:** Lifestyle risk factors.

Risk factors	*N* (%)
*Nutritional status*	
Obese	116 (9.7)
Overweight	156 (13)
Normoweight	928 (77.3)

*Physical activity*	
Less active	600 (50)
Active	600 (50)

*Perceived stress*	
Yes	382 (31.8)
No	818 (68.2)

*Sleep duration*	
Inadequate	940 (78.3)
Adequate	260 (21.7)

*Dietary intake sodium*	
High	80 (6.7)
Moderate	1120 (93.3)

*Fat*	
High	396 (33)
Moderate	804 (67)

*Fiber*	
Low	1192 (99.3)
Moderate	8 (0.7)

*Potassium*	
Low	1188 (99)
Moderate	12 (1)

*Calcium*	
Low	1176 (98)
Moderate	24 (2)

*Smoking*	
Yes	40 (3.3)
No	1160 (96.7)

*Smoking father*	
Yes	568 (47.3)
No	632 (52.7)

*Smoking mother*	
Yes	16 (1.3)
No	1184 (98.7)

**Table 3:** Blood pressure levels among adolescents.

Blood pressure levels	*N* (%)	Systolic BP (mmHg)	Diastolic BP (mmHg)
Normotension	958 (79.8)	109 ± 10.6	72 ± 8.5
Elevated blood pressure	146 (12.2)		
Grade I hypertension	84 (7)		
Grade II hypertension	12 (1)		

**Table 4:** Blood pressure levels according to sex characteristics.

Sex	Normotension	Elevated BP	Stage I HTN	Stage II HTN
Male	328 (27.3)	58 (4.8)	46 (3.8)	10 (0.8)
Female	630 (52.5)	88 (7.4)	38 (3.2)	2 (0.2)
Total	958 (79.8)	146 (12.2)	84 (7)	12 (1)

**Table 5:** Sociodemographic and lifestyle risk factors and hypertension among adolescents.

Variable	Hypertension		OR (95% CI)	*p* value
	Yes	No		
*Age group (years old)*				
Midadolescence (14 to 16)	84	910	1.49	0.432
Late adolescence (17 to 18)	12	194	(0.61–3.60)	

*Sex*				
Male	56	386	2.60	0.002^*∗*^
Female	40	718	(1.42–4.74)	

*Ethnicity*				
Palembang	68	804	0.90	0.738
Others	28	300	(0.47–1.73)	

*Family history of hypertension*				
Present	62	382	3.44	0.000^*∗*^
Absent	34	722	(1.86–6.38)	

*Hypertensive father*				
Yes	42	218	3.16	0.000^*∗*^
No	54	886	(1.72–5.80)	

*Hypertensive mother*				
Yes	30	218	1.84	0.065^*∗∗*^
No	66	886	(0.96–3.52)	

*Nutritional status*				
Obese/overweight	36	236	2.20	0.018^*∗*^
Normoweight	60	868	(1.18–4.09)	

*Physical activity*				
Less active	70	530	2.91	0.001^*∗*^
Active	26	574	(1.51–5.63)	

*Perceived stress*				
Yes	66	316	5.48	0.000^*∗*^
No	30	788	(2.90–10.3)	

*Sleep duration*				
Inadequate	76	864	1.05	1.000
Adequate	20	240	(0.51–2.18)	

*Dietary intake sodium*				
High	2	78	0.28	0.358
Moderate	94	1026	(0.03–2.08)	

*Fat*				
High	28	368	0.82	0.633
Moderate	68	736	(0.43–1.57)	

*Fiber*				
Low	96	1096	0.91	1.000
Moderate	0	8	(0.89–0.94)	

*Potassium*				
Low	96	1092	0.91	1.000
Moderate	0	12	(0.89–0.94)	

*Calcium*				
Low	96	1080	0.91	0.612
Moderate	0	24	(0.89–0.94)	

*Smoking*				
Yes	6	34	2.09	0.210^*∗∗*^
No	90	1070	(0.59–7.43)	

*Smoking father*				
Yes	52	516	1.34	0.367
No	44	588	(0.74–2.43)	

*Smoking mother*				
Yes	0	16	1.08	1.000
No	96	1088	(1.06–1.11)	

^∗^ *p* value < 0.05. ^∗∗^*p* value < 0.25.

**Table 6:** Final model of hypertension prevalence.

Variable	*B*	*p* value	OR (95% CI)	Nagelkerke *R* square
Sex	1.377	0.000	3.96 (2.01–7.80)	0.27
Family history of hypertension	0.994	0.004	2.70 (1.37–5.31)	
Nutritional status	0.787	0.028	2.19 (1.08–4.43)	
Physical activity	1.223	0.001	3.39 (1.66–6.93)	
Perceived stress	1.763	0.000	5.83 (2.91–11.6)	
Constant	−6.909	0.000	0.001	

## Data Availability

The datasets used and analyzed during the current study are available from the corresponding author on reasonable request.

## Abbreviations

AAP: American Academy of Pediatrics
ACC: American College of Cardiology
AHA: American Heart Association
BP: Blood pressure
CI: Confidence interval
HTN: Hypertension
OR: Odds ratio
PAQ-A: Physical Activity Questionnaire for Adolescents
PSS: Perceived Stress Scale
RDA: Recommended Dietary Allowances
WHO: World Health Organization.
